# Potent Activity of Venom from a Single Captive-Bred *Bitis rhinoceros* Specimen Against Planktonic Clinical MRSA and Early Biofilm Development

**DOI:** 10.3390/antibiotics15100984

**Published:** 2026-10-06

**Authors:** Marcos Hernando-Gozalo, Patricia Mayo-Magro, Carlos Rescalvo-Casas, Álvaro Camina-Vega, Laura Prieto-Pérez, Javier Durán-Camarón, Jordi Tena-Garcés, Juan J. Calvete, Emilio Mencías-Rodríguez, José Luis Méndez-García, Juan Cuadros-González, Ramón Pérez-Tanoira

**Affiliations:** 1 Clinical Microbiology Department, Príncipe de Asturias University Hospital, 28805 Alcalá de Henares, Madrid, Spain; m.hernando@uah.es (M.H.-G.); patrmayo@ucm.es (P.M.-M.); juan.cuadros@uah.es (J.C.-G.); 2 University of Alcalá, Department of Organic and Inorganic Chemistry, Faculty of Pharmacy, Research Institute in Chemistry ‘Andrés M. del Río’ (IQAR; UAH), Campus Universitario, 28805 Alcalá de Henares, Madrid, Spain; 3 University of Alcalá, Department of Biomedicine and Biotechnology, Faculty of Medicine, Campus Universitario, 28805 Alcalá de Henares, Madrid, Spain; 4 Grupo Atrox, Pabellón del Veneno, Faunia, 28032 Madrid, Spain; a.camina@grupoatrox.com (Á.C.-V.); jlmndez@yahoo.es (J.L.M.-G.); 5 ViberTOX, Grupo de Trabajo Especializado en el Tratamiento y Seguimiento de Mordeduras por Víboras en España (Clínica Humana y Veterinaria), Madrid, Spain; lprietope@fjd.es (L.P.-P.); emilio.mencias@justicia.es (E.M.-R.); 6 Internal Medicine Department, Fundación Jiménez Díaz and Faculty of Medicine, Autonomous University of Madrid, 28040 Madrid, Spain; 7 Master’s Degree in Tropical Medicine and International Health, Autonomous University of Madrid, 28049 Madrid, Spain; 8 University of Alcalá, Department of Systems Biology, Faculty of Medicine, Campus Universitario, 28805 Alcalá de Henares, Madrid, Spain; javier.duranc@edu.uah.es; 9 Evolutionary and Translational Venomics Laboratory, Instituto de Biomedicina de Valencia, Consejo Superior de Investigaciones Científicas (CSIC), 46010 Valencia, Spain; jtena@ibv.csic.es (J.T.-G.); jcalvete@ibv.csic.es (J.J.C.); 10 Centro Antitóxico, Instituto Nacional de Toxicología y Ciencias Forenses, 28232 Madrid, Spain

**Keywords:** antimicrobial resistance, *methicillin-resistant Staphylococcus aureus*, *Bitis rhinoceros* venom, biofilm formation inhibition, antimicrobial peptides, hemocompatibility, topical antimicrobial agents

## Abstract

**Background**: Antimicrobial resistance represents a major global health threat, particularly due to the increasing prevalence of multidrug-resistant pathogens such as methicillin-resistant *Staphylococcus aureus* (MRSA). Snake venoms have emerged as promising sources of bioactive molecules with potential antimicrobial applications. However, their activity against clinical MRSA isolates and bacterial biofilms remains insufficiently characterized. This study evaluated the antibacterial, biofilm formation inhibitory, and hemocompatibility properties of venom obtained from a captive-bred *Bitis rhinoceros* specimen against *S. aureus* strains and clinical MRSA isolates. **Methods**: Minimum inhibitory concentration (MIC), minimum bactericidal concentration (MBC), minimum biofilm inhibitory concentration (MBIC), minimum biofilm eradication concentration (MBEC), and biofilm formation inhibition assays were performed. In addition, direct and indirect hemolytic and hemagglutinating activities were evaluated to assess venom selectivity and biocompatibility. **Results**: Proteomic characterization revealed that the venom proteome is dominated by C-type lectin-like proteins (46.7%), with minor contributions from PIII snake venom metalloproteinase, phospholipase A_2_, and L-amino acid oxidase toxin families. *B. rhinoceros* venom exhibited potent bactericidal activity against planktonic *S. aureus* cells, with minimum inhibitory and bactericidal concentration values ranging from 4 to 16 mg/L. Notably, the two clinical MRSA isolates showed lower values than the reference strain, although this observation should be interpreted with caution given the limited number of isolates tested. The venom also strongly inhibited biofilm formation at the concentrations tested, with inhibition exceeding 97% and remaining statistically significant across all three strains and MIC multiples when analyzed at the level of the independent experiment (*n* = 3; paired *t*-test, Benjamini–Hochberg-adjusted *p* < 0.001), although limited activity was observed against preformed mature biofilms. Hemocompatibility assays demonstrated low direct and indirect hemolytic activity together with favorable selectivity indices against bacterial cells. **Conclusions**: These findings suggest that captive-bred *B. rhinoceros* venom may represent a promising source of antimicrobial compounds against planktonic MRSA infections and early-stage biofilm development. The favorable hemocompatibility profile supports its potential applicability as a topical antimicrobial candidate. Nevertheless, this study is a proof of concept and, therefore, further assays are required to isolate and functionally characterize the specific venom fractions responsible for the antimicrobial activity.

## 1. Introduction

Antimicrobial resistance (AMR) is currently one of the greatest global threats to public health, contributing substantially to morbidity, mortality, and healthcare costs worldwide [1,2,3,4]. Among multidrug-resistant pathogens, methicillin-resistant *Staphylococcus aureus* (MRSA) remains a major cause of persistent healthcare-associated and community-acquired infections, particularly skin, soft tissue, and device-related infections [3,4]. The increasing prevalence of antibiotic resistance highlights the urgent need for novel antimicrobial compounds with alternative mechanisms of action.

Biofilm formation further complicates the treatment of MRSA infections. Biofilms are structured microbial communities embedded within a self-produced extracellular polymeric matrix that protects bacteria from host immune responses and antimicrobial agents [5,6,7]. Consequently, biofilm-associated infections frequently exhibit increased antimicrobial tolerance and chronic persistence. Therefore, the identification of compounds capable of preventing biofilm formation or targeting resistant planktonic bacteria represents an important strategy in AMR research.

Snake venoms have emerged as promising sources of bioactive molecules with potential antimicrobial applications [8,9,10,11]. These venoms contain a wide variety of compounds, including phospholipases A_2_ (PLA_2_), L-amino acid oxidases (LAAO), metalloproteases, and antimicrobial peptides (AMPs), which may exert antibacterial effects through membrane disruption, enzymatic degradation, oxidative stress induction, and interference with biofilm development [10]. Antibacterial and anti-biofilm effects have been reported across multiple venom sources and toxin classes: LAAO from *Bothrops* and king cobra (*Ophiophagus hannah*) venoms impair biofilm formation in clinically relevant bacteria through hydrogen peroxide generation [12,13], while venom-derived peptides such as pseudonajide and AMPEC4 disrupt bacterial cell envelope integrity and inhibit the formation of biofilms by *Staphylococcus epidermidis* and *Escherichia coli*, respectively [14,15]. These two species differ substantially in cell-envelope architecture—the Gram-negative outer membrane of *E. coli* versus the Gram-positive peptidoglycan and wall-associated proteins (e.g., protein A, coagulase) of staphylococci—and no mechanistic equivalence with the *S. aureus*/MRSA data presented in this study should be inferred. Similarly, cationic peptides engineered from snake venom templates have shown broad-spectrum antimicrobial and disinfectant activity [16], and C-type lectin-like proteins, a structurally conserved toxin family across viperid venoms, have been associated with antibacterial and immunomodulatory functions [17]. Within the genus *Bitis* specifically, prior venomics work has demonstrated substantial intrageneric variation in venom toxin composition; Calvete et al., [18] characterized the venom proteome of *Bitis gabonica rhinoceros* (the taxonomic classification then in use for the present study’s specimen) and identified toxins spanning eleven distinct protein families, underscoring the compositional complexity that likely underlies the functional diversity of *Bitis* venoms. Furthermore, the study by Ouertani et al. [19] demonstrated the in vitro antibacterial activity of *B. gabonica rhinoceros*’ venom against the planktonic forms of a variety of bacteria. However, most previous antibacterial studies have evaluated venom activity using laboratory reference strains, whereas data regarding clinical MRSA isolates and biofilm-related activity remain limited. Therefore, the aim of this study was to evaluate the in vitro antibacterial, biofilm formation inhibitory, and hemocompatibility properties of venom obtained from a single traceable captive-bred *B. rhinoceros* specimen against clinical MRSA isolates and *S. aureus* reference strains. In addition, the study aimed to assess the therapeutic selectivity of the venom and its potential applicability as a topical antimicrobial candidate for AMR-associated infections.

## 2. Results

### 2.1. Antibacterial and Biofilm Formation Inhibitory Activity of Venom

The antibacterial and biofilm-related activities of *B. rhinoceros* venom against *S. aureus* strains are summarized in Table 1. The venom exhibited potent bactericidal activity against planktonic cells of all tested strains, with MIC and MBC values ranging from 4 to 16 mg/L. Notably, the two clinical MRSA isolates showed numerically lower MIC/MBC values than the American Type Culture Collection (ATCC) reference strain; however, no formal statistical comparison was performed between isolates given the small sample size (*n* = 2 clinical isolates), and this observation should be regarded as preliminary. In contrast, limited activity was observed against preformed mature biofilms, with MBIC and MBEC values exceeding the highest concentration tested (>1024 mg/L).

The venom also exhibited strong biofilm formation inhibitory activity against all tested *S.aureus* strains (Table 2). Biofilm formation inhibition exceeded 97% at all tested concentrations. To avoid pseudoreplication, statistical comparisons were performed using the independent experiment (*n* = 3) as the unit of analysis; treatment values remained significantly different from untreated controls at all concentrations and for all three strains (paired *t*-test, Benjamini–Hochberg-adjusted *p* ≤ 0.001; Table 2).

### 2.2. Hemocompatibility Properties of Venom

**Figure 1:**
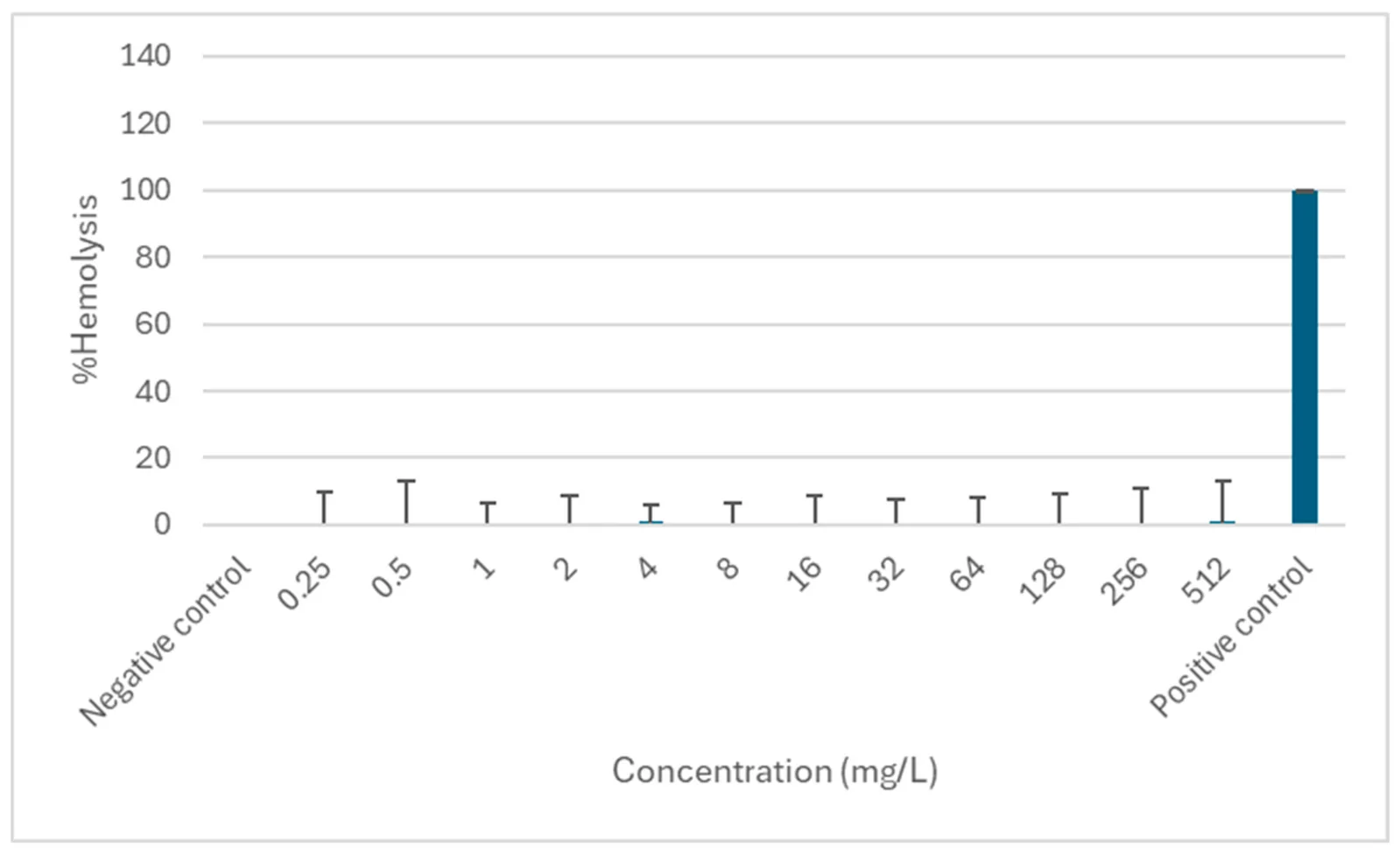
Direct hemolysis (absence of CaCl_2_) induced by *Bitis rhinoceros*’ venom. Triton X-100 20% and PBS were used as positive and negative controls for hemolysis, respectively.

**Figure 2:**
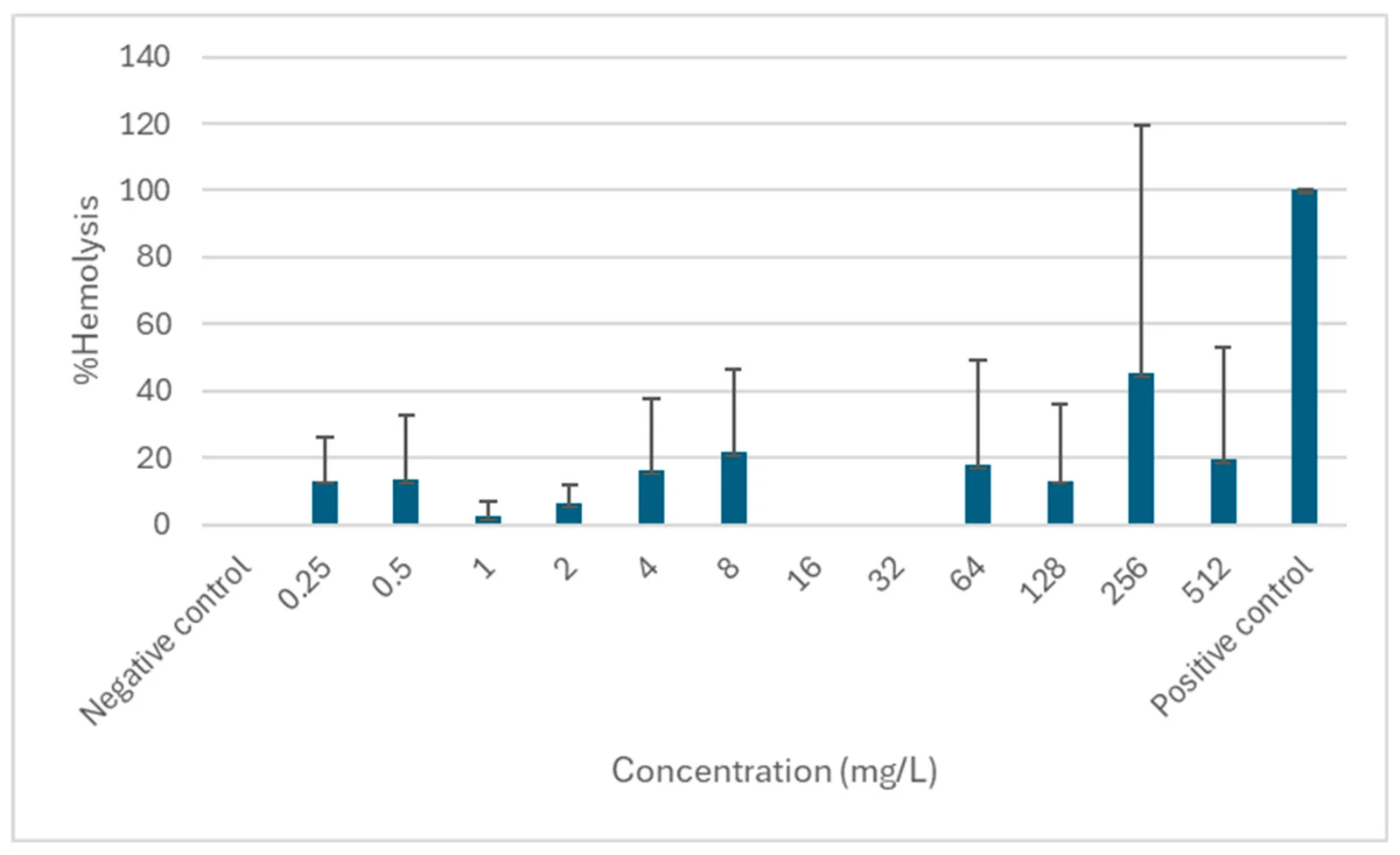
Indirect hemolysis (presence of CaCl_2_) induced by *Bitis rhinoceros*’ venom. Triton X-100 20% and PBS were used as positive and negative controls for hemolysis, respectively.

Hemolysis and hemagglutination assays were performed to evaluate venom hemocompatibility and selectivity toward eukaryotic cells. Direct hemolysis assays demonstrated low hemolytic activity in the absence of CaCl_2_, whereas indirect hemolysis levels increased in the presence of calcium (Figure 1 and Figure 2). Nevertheless, favorable selectivity indices (SI > 1) were observed for all tested strains, indicating greater selectivity toward bacterial cells than erythrocytes (Table 3). Statistically significant differences were observed compared with controls (*p* < 0.001).

**Figure 3:**
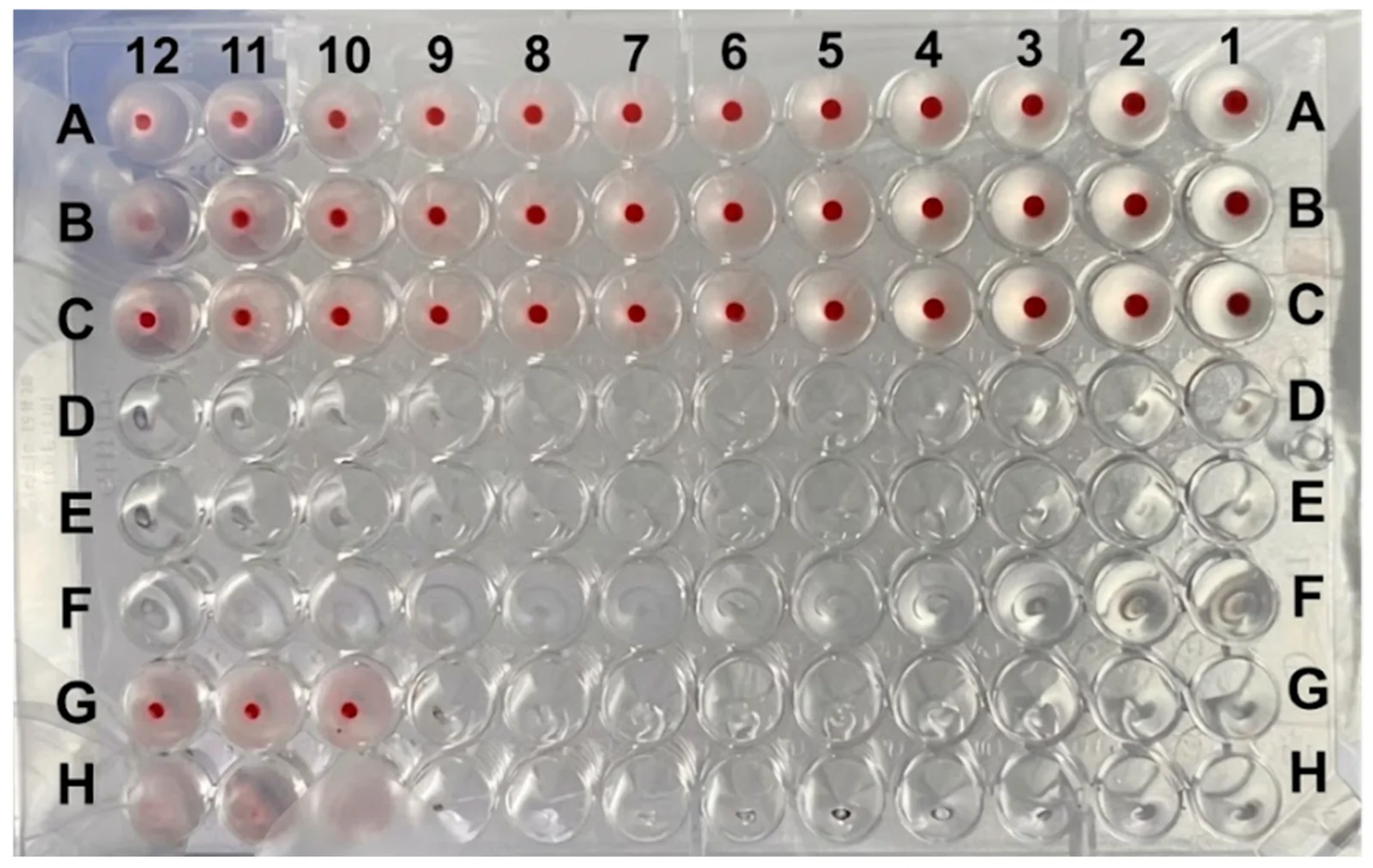
Direct hemagglutination assay of *Bitis rhinoceros* venom (without CaCl_2_). Rows A–C: venom concentrations (0.25–512 mg/L). Row D: negative venom control. Wells G10–G12 and H10–H12: negative and positive controls, respectively. Positive hemagglutination appears as a diffuse halo and negative results as a compact pellet at the bottom of the well.

**Figure 4:**
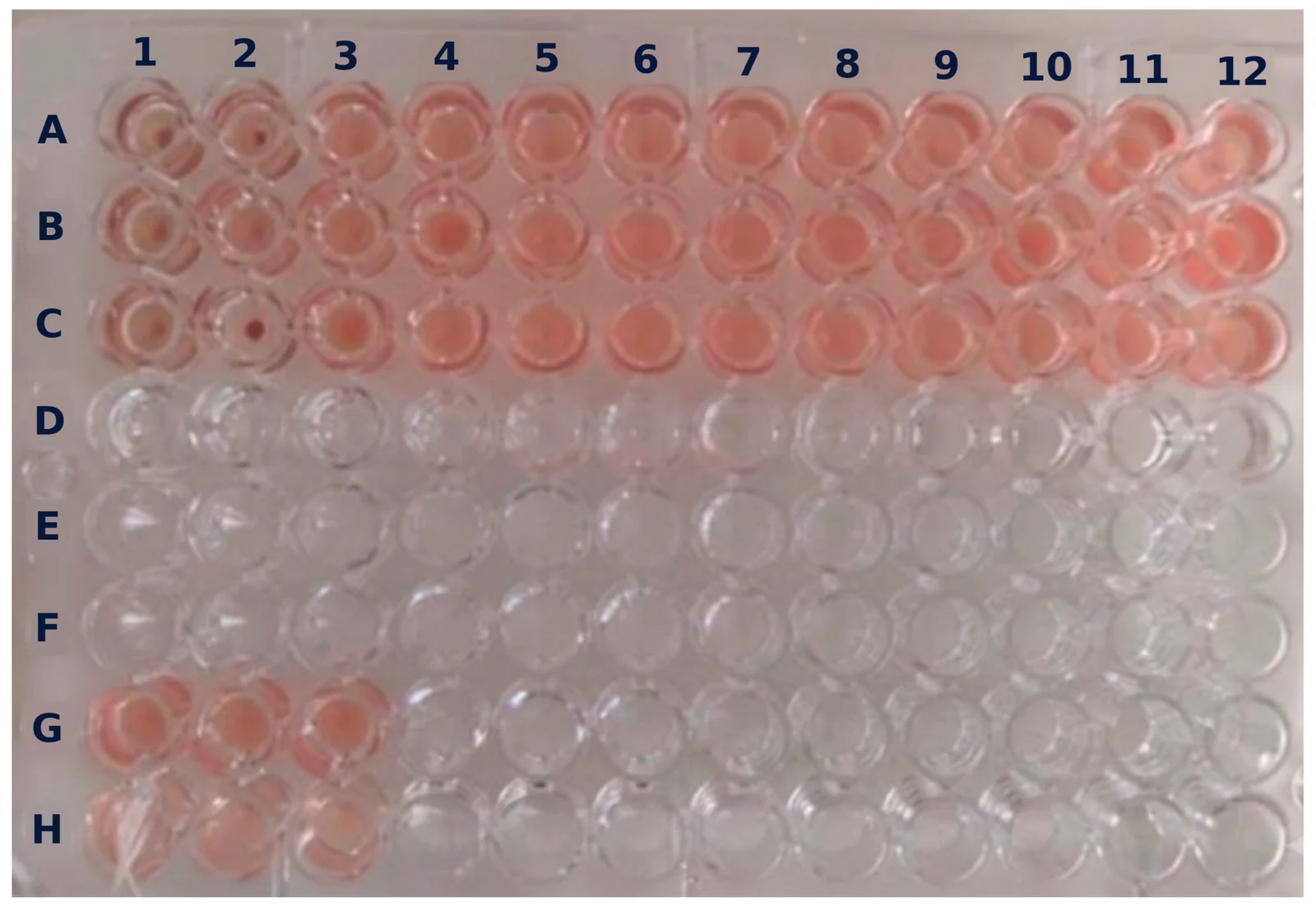
Indirect hemagglutination assay of *Bitis rhinoceros* venom (presence of CaCl_2_). Rows A–C: venom concentrations (0.25–512 mg/L). Row D: negative venom control. Wells G1–G3 and H1–H2: negative and positive controls, respectively.

No hemagglutination was observed in the absence of CaCl_2_, as a pellet was observed at the bottom of the well in all concentrations tested due to the sedimentation of erythrocytes (Figure 3). In contrast, indirect hemagglutination was observed in the presence of CaCl_2_, with a diffuse halo observed in the treated wells, which attenuated at higher concentrations (Figure 4).

### 2.3. Venom Proteome Composition

**Figure 5:**
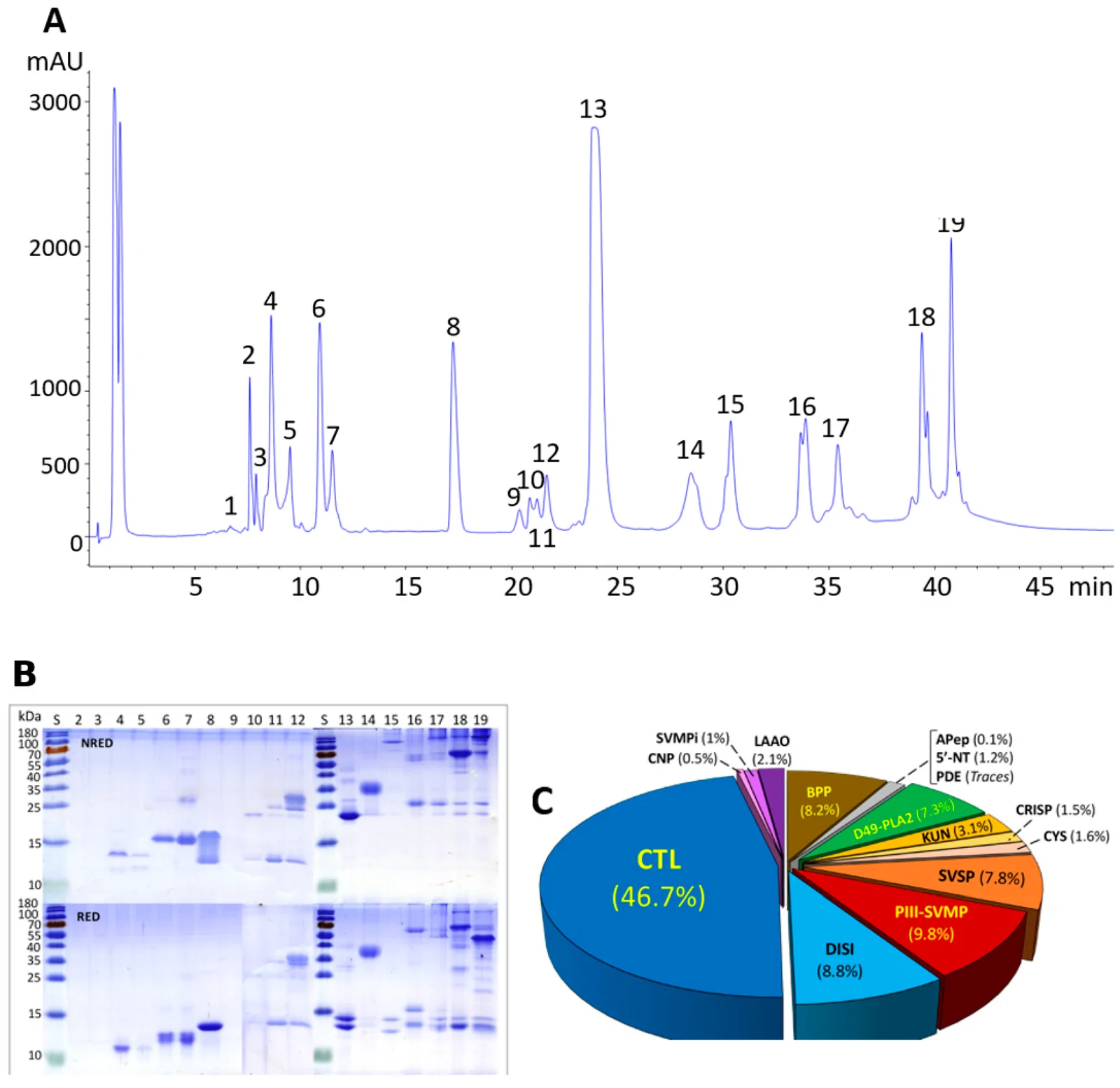
Decomplexation of the venom of a *Bitis rhinoceros* adult female by reversed-phase chromatography (**A**) followed by SDS-PAGE analysis of the chromatographic peaks in non-reduced (NRED) and reduced (RED) gels (**B**). The pie chart (**C**) displays the relative abundance of toxin families in the venom proteome as the ratio of the sum of the percentages of the individual proteins from the same toxin family (Supplementary Table S1). These relative abundance values were semi-quantitative. Values do not sum to exactly 100% due to rounding and trace components. Acronyms: CNP—C-type natriuretic peptide; SVMPi—tripeptide inhibitor of snake venom metalloproteinases (SVMPs); LAAO—L-amino acid oxidase; BPP—bradykinin-potentiating peptide; APep—aminopeptidase; 5′NT—5′-nucleotidase; PDE—phosphodiesterase; D49—catalytic aspartic acid 49; D49-PLA2—D49-phospholipase A2; KUN—Kunitz-type inhibitors; CRISP—cysteine-rich secretory protein; Cys—cystatin; SVSP—snake venom serine proteinase; PIII-SVMP—snake venom metalloproteinase of class III; DISI—disintegrin; CTL—C-type lectin-like.

Reversed-phase high-performance liquid chromatography (HPLC) fractionation followed by SDS-PAGE and mass spectrometry identified the toxin families comprising *B. rhinoceros* venom (Figure 5). These relative abundance values were semi-quantitative and the venom proteome was dominated by C-type lectin-like proteins (CTL, 46.7%), with minor contributions from PIII-SVMP (9.8%), PLA_2_ (7.3%), and LAAO (2.1%), among other low-abundance toxin families.

## 3. Discussion

This study demonstrated that venom obtained from a single captive-bred *B. rhinoceros* specimen exhibited potent bactericidal activity against planktonic *S. aureus* cells, including clinical MRSA isolates, together with strong biofilm-inhibitory activity. In contrast, only limited activity was observed against preformed mature biofilms. Additionally, the venom exhibited low hemolytic activity and favorable selectivity indices (SI = HC_50_/MIC [20]; SI ≥ 64–256 across the tested strains, Table 3), supporting its potential applicability as a source of topical antimicrobial compounds. Regarding the antibacterial activity of the venom against planktonic forms of *S. aureus*, it showed potent bactericidal activity against these bacteria, consistent with previous reports [9,11]. Sulca et al. [9] demonstrated that crude venom and peptide fractions from *Bothriopsis oligolepis* inhibited multiple pathogens, with superior efficacy against *S. aureus*. Similarly, Sudarshan et al., [11] reported that NN-XIb-PLA_2_ (acidic phospholipase A_2_ fraction-XIb) from *Naja naja* venom exhibited bactericidal activity against *S. aureus* and other bacteria. Although the MIC values observed for *B. rhinoceros* venom (4–16 mg/L) remain markedly higher than those typically reported for conventional anti-MRSA agents against MRSA clinical isolates, e.g., vancomycin (MIC_50_/_90_ = 1/1–2 mg/L) [21], linezolid (MIC_50_/_90_ = 1/2 mg/L) [21], and mupirocin (MIC_50_/_90_ = 0.25/1 mg/L) [22]—the activity observed for a crude, unfractionated venom preparation remains notable and supports further fractionation studies to isolate and characterize the most potent antimicrobial components. Interestingly, the venom showed numerically lower MIC/MBC values against the two clinical MRSA isolates than against the ATCC reference strain. However, this observation is based on only two clinical isolates from a narrow patient and infection profile, without molecular typing (e.g., spa-typing or Multilocus Sequence Typing (MLST)) to determine their genetic relatedness; the observed MIC difference (4 mg/L vs. 16 mg/L) may therefore reflect strain-specific variation rather than a generalizable trend, and this finding should be considered preliminary and hypothesis-generating rather than conclusive. This finding highlights the importance of evaluating novel antimicrobial compounds using clinically relevant isolates rather than relying exclusively on laboratory strains, which may not fully reproduce the phenotypic and virulence characteristics observed in real-world infections. Differences in susceptibility may be associated with strain-specific variations in cell wall composition, virulence determinants, or membrane-associated factors.

The venom exhibited strong biofilm formation inhibitory activity against all tested *S. aureus* strains but only limited activity against preformed mature biofilms. The preventive effect may be associated with venom components such as phospholipases A_2_, L-amino acid oxidases, and antimicrobial peptides, which can compromise bacterial membrane integrity and interfere with early biofilm development [10]. Similar findings have been reported for venom-derived compounds from *Bothrops* spp. and *O. hannah*, which significantly inhibited *S. aureus* biofilm formation [12,13]. It should be noted that biofilm formation inhibition was evaluated only at concentrations at or above the MIC (1×, 2×, and 4× MIC); consequently, the near-complete inhibition observed even at the lowest tested concentration may partly reflect direct bactericidal activity rather than a biofilm-specific preventive mechanism. Future studies including sub-inhibitory (sub-MIC) concentrations would help determine whether the venom retains anti-biofilm activity independent of its bactericidal effect. In contrast, some synthetic venom-derived peptides have demonstrated partial eradication of mature MRSA biofilms [16], suggesting that activity against established biofilms may depend on the biochemical composition and physicochemical properties of the active fractions. Therefore, *B. rhinoceros* venom may be more suitable for infection prevention, early-stage biofilm control, or topical antimicrobial applications rather than eradication of mature biofilms.

It should be noted that the relative abundances shown in Figure 5C derive from a single RP-HPLC decomplexation of venom pooled from one specimen, without technical or biological replicates; therefore, these values should be interpreted as representative of this individual venom sample rather than as a fully validated quantitative profile of the species, and inter-individual variability cannot be excluded.

The pronounced CTL dominance (46.7%) observed in the present study contrasts with prior proteomic characterizations of *Bitis* venoms. Nguyen et al. [23], in a systematic proteomic survey of 26 medically important sub-Saharan African elapid and viper venoms, reported that *B. rhinoceros* venom is instead dominated by PLA_2_ (39%) and SVSP (18%), with comparatively low CTL content. Similarly, Calvete et al., [24] found that the venom of the closely related taxon *Bitis gabonica gabonica* is dominated by SVSP (26.4%) and SVMP (22.9%), with CTLs representing a more modest 14.3% of the proteome. This discrepancy may reflect genuine inter-individual and/or captive-versus-wild venom variability—consistent with the single-specimen limitation already acknowledged in this study—rather than a taxon-wide compositional trait, and underscores the need for venom proteomic studies incorporating multiple specimens to establish representative compositional profiles for this species. The venomic characterization performed in this study provides additional mechanistic insight into the biological activities observed. C-type lectin-like proteins (CTLs) represented the predominant toxin family in the venom proteome (46.7%), which may explain the calcium-dependent hemagglutinating activity observed in the presence of CaCl_2_, consistent with the known ability of CTLs to interact with platelet and erythrocyte membrane receptors [17]; however, as CTLs are typically non-enzymatic, a direct bactericidal mechanism for this fraction is mechanistically less parsimonious. In addition, the presence of phospholipases A_2_ (PLA_2_s) and L-amino acid oxidases (LAAOs), both previously associated with membrane disruption, oxidative stress induction, and antibacterial activity, may contribute more directly to the potent activity observed against planktonic MRSA isolates [10,11], potentially acting in synergy despite their comparatively minor relative abundance. Conversely, the relatively high abundance of PIII-snake venom metalloproteinases (SVMPs) may partially explain the cytotoxic and hemocompatibility limitations observed, as these toxins are known to induce tissue damage and affect mammalian cell integrity. Given the cytotoxic potential associated with SVMPs, we do not propose the crude venom itself as a clinical topical agent; rather, these findings support prioritizing purified or recombinant components for future functional testing. In particular, we hypothesize that the PLA_2_ and LAAO fractions may be the primary contributors to the antibacterial and anti-biofilm activity observed, with the CTL fraction representing a comparatively more speculative candidate given its typically non-enzymatic nature; however, this remains to be functionally validated, as individual toxin fractions were not tested in isolation in the present study, while excluding or minimizing the SVMP-rich fractions responsible for cytotoxicity. Together, these findings support a multifactorial mechanism underlying the antimicrobial and biological activities of *B. rhinoceros* venom. In addition to antimicrobial activity, evaluation of venom hemocompatibility is essential to determine its potential translational applicability, showing greater selectivity toward bacterial cells than erythrocytes. The venom exhibited favorable selectivity indices together with low hemolytic activity (SI > 1) with low levels of direct and indirect hemolysis, as well as calcium-dependent hemagglutination. The low levels of direct and indirect hemolysis observed in this study are consistent with previous reports indicating that crude snake venoms may exhibit lower hemolytic activity than isolated purified fractions [8]. Similar findings have been described for several venom-derived antimicrobial peptides displaying potent antibacterial and biofilm inhibitory activity together with favorable hemocompatibility profiles [15,16]. Interestingly, hemagglutination was only observed in the presence of CaCl_2_, suggesting the possible involvement of calcium-dependent lectin-like venom components [17].

The non-monotonic hemolysis pattern observed in the indirect hemolysis assay (Figure 2), including the wide variability at 256 mg/L and the apparent reduction in mean hemolysis at 512 mg/L relative to 256 mg/L, may reflect venom-induced erythrocyte aggregation, rather than a genuine reduction in hemolytic activity. This is consistent with the calcium-dependent hemagglutinating activity independently observed in this study (Figure 4) and with reports of CTL-mediated erythrocyte aggregation in other *Bitis venoms*, which can promote platelet receptor binding and fibrinogen association. Because absorbance was measured in the post-centrifugation supernatant (3500 rpm, 15 min; Section 4.7), we consider it more likely that hemoglobin released at high venom concentrations became partially entrapped within sedimented erythrocyte/debris aggregates during centrifugation, rather than free hemoglobin in the supernatant itself scattering light, thereby reducing the hemoglobin quantified in the cleared supernatant; no additional spectroscopic correction was applied to account for this potential effect. All *n* = 3 independent replicates were included in the analysis at each concentration, with no data exclusions.

Furthermore, at the lowest venom concentrations, the modest hemolytic responses may reflect the combined effects of low-level biological activity and the greater relative impact of experimental variability when absorbance values are close to the assay detection limit. The absence of detectable hemolysis at intermediate venom concentrations cannot be conclusively explained by the present data. As crude snake venoms comprise multiple bioactive components with distinct and potentially overlapping mechanisms of action, non-linear concentration–response relationships may arise from concentration-dependent interactions among these components. Consequently, the non-monotonic profile likely reflects the complex composition of the crude venom, together with analytical interference caused by erythrocyte aggregation at higher venom concentrations.

The potential of *B. rhinoceros* venom as a candidate source for topical antimicrobial development is supported by three converging lines of evidence from the present study, considered alongside established criteria from the antimicrobial peptide/compound literature. First, the venom exhibited potent bactericidal activity against clinically relevant MRSA isolates (MIC 4–16 mg/L) and strongly inhibited early biofilm formation (>97% inhibition), both of which are directly relevant to the prevention of skin and wound infections. Second, the venom’s hemocompatibility profile (low hemolytic activity, SI ≥ 64–256; Table 3) exceeds the SI > 10 threshold conventionally regarded as indicative of a favorable safety margin for topical or external antimicrobial applications in the antimicrobial peptide development literature [20,25]. Third, this profile is consistent with precedents in the field, where other snake venom-derived peptides and crude fractions have been specifically proposed for topical, wound-healing, or disinfectant applications based on comparable potency and selectivity profiles [16]. Nonetheless, we acknowledge that these criteria support only a preliminary, hypothesis-generating case for topical applicability; formal translational validation (e.g., ex vivo skin models, cytotoxicity against human keratinocytes/fibroblasts, and stability/formulation studies) was beyond the scope of this exploratory study and represents a necessary next step.

This study has several limitations related to the source of the venom, specifically its sex, age, diet, captive husbandry, and overall representativeness. Firstly, venom was obtained from a single adult female captive-bred specimen, which limits extrapolation of the findings to the species as a whole. Regarding sex-specific effects, sex-based venom variation is well documented in other viperid genera such as *Bothrops* [26], but no published data on sex-based venom variation exist for *B. rhinoceros* or closely related *Bitis* taxa; the only available intraspecific study within the genus (a litter of *B. gabonica*) found individual electrophoretic variation but no sex-based differences [27]. Therefore, whether the findings reported here reflect a general species-level trait or a female-specific venom profile cannot be established, and this should not be assumed to be equivalent to male venom composition. Secondly, regarding captive husbandry, although captive breeding provides traceability, no published comparison of captive-versus-wild *B. rhinoceros* venom composition was located, and the degree to which this specimen’s venom is representative of wild conspecifics remains unknown; captive-bred venom should therefore not be assumed equivalent to wild-type venom for this species. Thirdly, this study was designed as a proof-of-concept trial in which only three bacterial strains were used, which limits the possibility of drawing more definitive conclusions. Consequently, it would be highly advisable to test the venom on a larger number of both reference and clinical strains in order to better establish its therapeutic potential. Fourthly, the comparison performed in this study confounds strain origin with methicillin-resistance status, since the reference strain (ATCC 29213) is methicillin-susceptible while both clinical isolates are MRSA; the numerically lower MIC/MBC values observed for the clinical isolates therefore cannot be attributed to their clinical origin alone, and may instead reflect resistance-associated phenotypic differences. A properly controlled comparison would require an additional methicillin-resistant reference strain and/or methicillin-susceptible clinical isolates to disentangle these two variables. Fifthly, MIC and MBC determinations were performed under 5% CO_2_ incubation rather than the ambient-air condition specified by CLSI M07 guidelines [28,29], due to equipment availability constraints; because Müller–Hinton broth is not a buffered medium, CO_2_-induced acidification may have had a more pronounced effect on bacterial growth kinetics and on the activity of pH-sensitive venom components than it would in a buffered system, and the extent to which this deviation influenced the absolute MIC/MBC values reported here cannot be fully excluded, and confirmatory testing under standard conditions is warranted in future work. Additionally, the biofilm formation inhibition analysis relies on only three independent experiments per strain and concentration; although the effect was large and highly consistent across all three experiments (allowing paired parametric testing to reach statistical significance, Table 2), the number of independent replicates remains small, and future studies should increase it to allow more robust modelling of the data. Sixthly, cytotoxicity was only evaluated using erythrocyte-based assays, and additional mammalian cell models should be investigated. Finally, the active venom fractions responsible for the observed antimicrobial activity were not characterized. Future studies should address these gaps through venom fractionation, sampling of additional specimens (including males and wild-caught individuals where feasible), evaluation of additional cytotoxicity models, and optimization of strategies targeting mature biofilms.

## 4. Materials and Methods

### 4.1. Venom Extraction and Lyophilization

Crude venom was manually “milked” from an adult female *B. rhinoceros* specimen (bred in captivity at Faunia, Madrid, Spain) over multiple sessions between October and November 2024. Venom extraction was performed manually using a paraffin-coated plastic container (~2–2.5 mL per session) and was spaced at intervals of at least 23 days to allow adequate venom gland regeneration, following the feeding and recovery protocol certified by Faunia’s Internal Animal Welfare Committee. Venom from each session was collected via insulin syringe, transferred to a 14 mL Falcon™ tube (Thermo Fisher Scientific, Waltham, MA, USA), and transported on ice to the laboratory, where samples were frozen at −20 °C. Pooled venom from all extraction sessions was subsequently lyophilized together using a Telstar Cryodos-50 freeze-dryer (Terrassa, Spain), yielding a total of 1.1322 g of pure, traceable powder stored at −20 °C. The use of captive-bred venom, obtained under a controlled and documented extraction schedule, ensured full traceability of the material used throughout the study.

### 4.2. Characterization of the B. rhinoceros Venom Proteome

#### 4.2.1. Reversed-Phase and SDS-PAGE Profiling of B. rhinoceros Venom

Two hundred and fifty micrograms of *B. rhinoceros*’ venom were dissolved in 50 μL of 0.05% trifluoroacetic acid (TFA) and 5% acetonitrile (ACN). Insoluble material was removed by centrifugation in an Eppendorf centrifuge (Eppendorf SE, Hamburg, Germany) at 13,000× *g* for 10 min at room temperature, and the proteins contained in 40 μL were separated by RP-HPLC using an Agilent LC 1100 High-Pressure Gradient System (Agilent Technologies, Santa Clara, CA, USA) equipped with a Discovery^®^ BIO Wide Pore C18 (15 cm × 2.1 mm, 3 μm particle size, 300 Å pore size) column (Supelco, Bellefonte, PA, USA) and a DAD detector. The column was developed at a flow rate of 0.4 mL/min with a linear gradient of 0.1% TFA in MilliQ^®^ water (Merck Millipore, Burlington, MA, USA) (solution A) and 0.1% TFA in acetonitrile (solution B), isocratic (5% B) for 1 min, followed by 5–25% B for 5 min, 25–45% B for 30 min, and 45–70% B for 5 min. Protein detection was carried out at 215 nm with a reference wavelength of 400 nm. Fractions were collected manually across the entire elution range, dried in a vacuum centrifuge (Savant™, Thermo Scientific Inc., West Palm Beach, FL, USA), redissolved in MilliQ^®^ water, and analyzed by SDS-PAGE on 12 or 15% polyacrylamide gels. To this end, sample aliquots were mixed with ¼ volume of 4 × sample buffer (0.25 M Tris-HCl pH 6.8, 8% SDS, 30% (*v*/*v*) glycerol, 0.02% (*w*/*v*) bromophenol blue, without or with 1% 2-mercaptoethanol), heated at 65 °C for 5 min, and run under non-reducing and reducing conditions. Gels were stained with colloidal Coomassie Brilliant Blue G-250 (A3480, PanReac, Barcelona, Spain) at a final concentration of 80 mg/L in distilled water containing 35 mM hydrochloric acid (HCl), for 1 h at room temperature under gentle agitation, and subsequently destained with distilled water (with several changes over 12–24 h) until adequate background clearance was achieved.

#### 4.2.2. Mass Spectrometric Identification of the Reversed-Phase and SDS-PAGE Resolved B. rhinoceros Venom Proteins

Protein bands excised from Coomassie Brilliant Blue-stained SDS-PAGE gels of the chromatographic fractions collected through RP-HPLC venom decomplexation were subjected to automated in-gel reduction (10 mM dithiothreitol) and alkylation (50 mM iodoacetamide), followed by overnight sequencing-grade trypsin digestion (66 ng/μL in 25 mM ammonium bicarbonate, 10% acetonitrile; 0.25 μg/sample) in an automated ProGest Protein Digestion Workstation (Genomic Solution Ltd., Cambridgeshire, UK). The tryptic digests were dried in a SpeedVac (Savant^TM^, Thermo Scientific Inc., West Palm Beach, FL, USA), redissolved in 15 μL of 0.1% formic acid in water, and submitted to LC-MS/MS. To this end, tryptic peptides were separated by nano-Acquity UltraPerformance LC (UPLC) using BEH130 C18 (100 μm × 100 mm, 1.7 μm particle size) column in-line with a SYNAPT G2 High-Definition Mass Spectrometry System (Waters Corp. Milford, MA, USA). The flow rate was set to 0.6 μL/min and the column was developed with a linear gradient of 0.1% formic acid in water (solution A) and 0.1% formic acid in acetonitrile (solution B), isocratically 1% B for 1 min, followed by 1–12% B for 1 min, 12–40% B for 15 min, 40–85% B for 2 min. Doubly and triply charged ions were selected for collision-induced dissociation (CID) MS/MS. Fragmentation spectra were interpreted (a) manually (de novo sequencing); (b) using the online form of the MASCOT program at http://www.matrixscience.com against the NCBI non-redundant database; and (c) processed in Waters Corporation’s ProteinLynx Global SERVER 2013 version 2.5.2 (with Expression version 2.0). The fragment mass tolerance (0.6 Da) applied in this study is consistent with established practice in venom proteomics using Q-TOF instrumentation and MASCOT-based database searching, and was complemented by manual de novo sequence verification to minimize false-positive identifications.

The precursor mass tolerance was set to 1.2 Da (MASCOT default setting). A tolerance of this magnitude is historically standard for ion trap and older Q-TOF instruments, and is occasionally applied in broader searches to ensure that +1 Da ^13^C precursor isotope peaks are captured when monoisotopic picking fails. Although a looser precursor tolerance increases the number of candidate peptides tested by MASCOT within the error window, it does not affect the Ions Score, which is determined entirely by MS/MS fragment ion matches. All peptide-spectrum matches were manually validated, and ions of ambiguous or low-confidence assignment were excluded, since a larger number of matched ions does not by itself improve the confidence of protein identification. Carbamidomethyl cysteine and oxidation of methionine were selected as fixed and variable modifications, respectively. Amino acid sequence similarity searches were performed against the available databanks using the BLAST program implemented in the WU-BLAST2, version 2.0 (Washington University, St. Louis, MO, USA) search engine at http://www.bork.embl-heidelberg.de.

#### 4.2.3. Relative Quantification of the Toxin Families in the Venom Proteome

The relative abundances of the chromatographic peaks obtained by reverse-phase HPLC fractionation of the whole venom were calculated as “% of total peptide bond concentration in the peak” by dividing the peak area by the total area of the chromatogram (Supplementary Table S1). For chromatographic peaks containing single components (as judged by SDS-PAGE and/or MS), this value is a good estimate of the % by weight (g/100 g) of the pure venom component [30,31]. When more than one venom protein was present in a reverse-phase fraction, their proportions (% of total protein band area) were estimated by densitometry of Coomassie-stained SDS–polyacrylamide gels using MetaMorph^®^ Image Analysis Software, version 7.10.5 (Molecular Devices, LLC, San Jose, CA, USA). Conversely, the relative abundances of different proteins contained in the same SDS-PAGE band were estimated based on the relative ion intensities of the three most abundant peptide ions associated with each protein by MS/MS analysis. The relative abundances of the protein families present in the venom were calculated as the ratio of the sum of percentages of the individual proteins from the same toxin family to the total area of venom protein peaks in the reverse-phase chromatogram, as described [32].

### 4.3. Bacterial Strains

Antimicrobial testing targeted biofilm-forming *S. aureus* ATCC^®^ 29213™ and two clinical MRSA isolates (MRSA 1 and MRSA 2) from Jiménez Díaz Foundation University Hospital (Madrid, Spain) [33]. MRSA 1 was isolated from a 73-year-old male’s infected wound and MRSA 2 from a 92-year-old male’s paronychia. Both isolates were confirmed as biofilm-forming strains. The simultaneous use of both antibiotic-sensitive and antibiotic-resistant strains enabled us to determine whether the venom was effective against both types of strain. These were stored at −80 °C, revived on colistin-nalidixic acid (CNA) agar and incubated at 37 °C in 5% CO_2_ for 24 h.

### 4.4. MIC and MBC Determination

The minimum inhibitory concentration (MIC) was determined based on Clinical and Laboratory Standards Institute (CLSI) M07 broth microdilution methodology (11th ed., 2018) [28], with modifications. Bacterial suspensions were prepared in saline at ~1.6 × 10^8^ Colony Forming Units per millilitre (CFU/mL), corresponding to 0.50 ± 0.02 on the McFarland turbidity scale. These were then diluted 1:100 in Müller-Hinton broth (MHB), yielding a final working inoculum concentration of ~1.6 × 10^6^ CFU/mL in the microdilution plate. Two-fold serial venom dilutions (1–1024 mg/L; 11 concentrations) and inoculum were added to 96-well round-bottom plates (Thermo Fisher Scientific, Roskilde, Denmark). Plates were incubated statically at 37 °C for 24 h. Incubation was performed under 5% CO_2_ rather than the ambient-air condition specified by CLSI M07 for standard broth microdilution testing of *S. aureus*; CLSI and EUCAST guidelines recommend CO_2_-enriched incubation specifically for fastidious organisms [29,34], a category that does not include *S. aureus*. A CO_2_ incubator was used by default in this study, as our laboratory does not have any other type available; the potential impact of this deviation is discussed in Section 3 (Limitations). MICs were determined by measuring absorbance at 600 nm on a plate reader Epoch™ (BioTek Instruments, Winooski, VT, USA).

The MIC was defined as the lowest venom concentration producing ≥80% reduction in OD_600_ relative to the drug-free growth control well, after subtraction of the corresponding venom-only (no-inoculum) background absorbance. This spectrophotometric threshold was used as a quantitative surrogate for the visually read MIC endpoint specified in CLSI M07 [28], since bacterial growth inhibition in this study was assessed instrumentally by absorbance rather than by unaided-eye turbidity inspection. Venom dilutions without bacterial inoculum were included as negative absorbance controls. The positive control in the experiment consisted of broth plus bacteria, whilst the negative control was sterile broth. The final volume was 200 µL of broth: 100 µL of venom at the specific concentration and 100 µL of bacterial inoculum at a concentration of 1.6 × 10^6^ CFU/mL.

The minimum bactericidal concentration (MBC) was assessed via flash microbicidal method [35]. From MIC plates, 20 µL/well were subcultured into 180 µL of Tryptic-Soy Broth (TSB) in 96-well flat-bottom plate, which was statically incubated at 37 °C and 5% of CO_2_ for 24 h. MBC values were determined by measuring absorbance at 600 nm using a microplate reader; the MBC was defined as the lowest venom concentration at which the subcultured well showed ≥90% reduction in OD_600_ relative to the growth control, consistent with the conventional operational threshold for bactericidal (≥99.9% killing) activity. All assays performed in quadruplicate (*n* = 4).

### 4.5. MBIC and MBEC Assays

After analyzing the antimicrobial properties of the venom against the planktonic forms of *S. aureus*, its efficacy against their biofilms was evaluated. On the one hand, it was tested against established biofilms using the minimum inhibitory biofilm concentration (MBIC) and minimum eradication biofilm concentration (MBEC) assays; on the other hand, its preventive activity in the early stages of biofilm formation was analyzed using the biofilm formation inhibition assay, which is detailed in the following section.

The minimum biofilm inhibitory concentration (MBIC) is the minimum concentration of an antimicrobial required to inhibit the visible growth of a bacterial biofilm, while the minimum biofilm eradication concentration (MBEC) is the minimum concentration of an antimicrobial needed to kill a bacterial biofilm [36]. MBIC and MBEC were determined using Pettit et al.’s protocol [37], adapted for non-motile *S. aureus*.

For MBIC of venom, biofilm formation was induced at the bottom of 96-well flat-bottom plates by adding 100 µL/well of MHB at a concentration of 1.6 × 10^6^ CFU/mL. This plate was incubated statically at 37 °C and 5% of CO_2_ for 24 h. Subsequently, the supernatant was aspirated from the wells and 200 µL/well of the venom dilutions (1–1024 mg/L) in MHB were added. This plate was incubated statically at 37 °C in 5% CO_2_ for 24 h, and biofilm viability after venom exposure was determined by adding 20 µL/well at a concentration of 5 mg/mL of 3-(4,5-dimethylthiazol-2-yl)-2,5-diphenyltetrazolium bromide (MTT) (Thermo Scientific, Kandel, Germany) at a concentration of 5 mg/mL and subsequent incubation at 37 °C for 0.5 h. The MBIC was obtained by measuring the absorbance at 570 nm in a plate reader.

For the MBEC, with the help of a 100 µL pipette, the bottom of the MBIC plate was scraped and 20 µL was collected, then subcultured in a new 96-well flat-bottom plate to which 180 µL of TSB had previously been added. This plate was incubated statically at 37 °C and 5% of CO_2_ for 24 h, and the MBEC was determined by directly measuring the absorbance at 600 nm in a plate reader. All experiments were carried out in quadruplicate (*n* = 4).

### 4.6. Inhibition of Biofilm Formation

These experiments were specifically performed to assess the preventive activity of the venom during early biofilm development and were performed according to the protocol of Pettit et al., with slight modifications [38]. To this end, an overnight culture of each strain was prepared and incubated at 37 °C and 5% CO_2_. The culture was then centrifuged at 3500 revolutions per minute (rpm) for 10 min and washed at least once with 0.9% saline solution. After resuspension in serum, it was diluted to a bacterial concentration of 1.6 × 10^8^ CFU/mL, equivalent to 0.50 ± 0.02 on the McFarland turbidity scale. In a 96-well flat-bottom plate, 200 µL/well of 1.6 × 10^6^ CFU/mL in TSB supplemented with 1% glucose, with or without venom (positive control), was added at the respective concentrations of 4 × MIC, 2 × MIC, and 1 × MIC for each strain. These concentrations were selected based on previous studies evaluating antimicrobial activity against biofilm-forming bacteria [39]. This plate was incubated statically at 37 °C and 5% CO_2_, and biofilm formation inhibition was quantified by directly measuring the absorbance at 595 nm in a plate reader. This measurement was accompanied by a series of double dilutions of the venom as a negative absorbance control. These experiments were performed in eight technical replicate wells per concentration, across three independent experiments per strain (biological triplicate), yielding a total of *n* = 24 replicates per concentration and strain.

### 4.7. Hematological Assays

Hemolysis was used to assess the toxicity of the venom on erythrocyte membranes by detecting their rupture and the release of hemoglobin into the extracellular medium. These assays were performed to evaluate venom hemocompatibility and selectivity toward eukaryotic cells. Moreover, it was performed in accordance with ISO 10993-4 protocol [40] both in the absence of CaCl_2_ (direct hemolysis) and in the presence of CaCl_2_ (indirect hemolysis) because the enzymatic activity of certain hemolytic components of the venom, such as PLA_2_, may depend on the presence of the Ca^2+^ ion [41].

First, with respect to direct hemolysis, an erythrocyte suspension was prepared by centrifuging 2 mL of defibrinated sheep blood (Oxoid Thermo Scientific, Basingstoke, UK) at 3500 rpm for 10 min. The supernatant was then discarded, and the resulting pellet (erythrocytes) was resuspended in 2 mL of phosphate-buffered saline (PBS, pH = 7.4) and centrifuged under the same conditions. This washing process was repeated three more times to remove serum proteins and plasma residues. After washing, the sediment was resuspended in 2 mL of PBS and a 1:50 erythrocyte suspension was prepared in PBS, which was incubated at 37 °C for 15 min. A series of two-fold serial venom dilutions in PBS (12 concentrations) were carried out in a concentration range of 2.5–5120 mg/L. In 1.5 mL Eppendorf tubes, these solutions were diluted tenfold, yielding a final concentration range of 0.25–512 mg/L (12 concentrations). This difference in the number of concentrations tested between assays is not due to any specific methodological reason; both ranges were considered appropriate for their respective assay. Then, 180 µL of the 1:50 erythrocyte suspension and 20 µL of each concentration to be analyzed were added, and the mixture was incubated for 2 h at 37 °C. The Eppendorf tubes were then centrifuged at 3500 rpm for 15 min, and 200 µL of the supernatant from each tube was transferred to a 96-well flat-bottom plate. Hemolysis was determined by quantifying hemoglobin release by directly measuring absorbance at 540 nm in a plate reader. This experiment was accompanied by a series of negative venom controls, mixing 180 µL of PBS and 20 µL of each concentration. In addition, a positive control and a negative control for hemolysis were prepared in the same manner, using Triton X-100 20% and PBS, respectively. These experiments were performed in triplicate (*n* = 3).

The percentage of hemolysis was calculated using the following formula: %Hemolysis = [(A_S_-A_VC_)/(A_PC_-A_NC_)] × 100, where A_S_, A_VC_, A_PC_, and A_NC_ are the absorbances of the sample, the negative venom control, the positive control, and the negative hemolysis control, respectively. The 50% hemolytic concentration (HC_50_), which is the concentration of a compound necessary to induce 50% hemolysis of the erythrocyte population in vitro, was calculated using the Quest Graph™ EC50 Calculator (AAT Bioquest, Inc., Pleasanton, CA, USA) [42].

Finally, the selectivity of the venom against microorganisms was calculated using the selectivity index (SI) [20], whose formula is as follows: SI = HC_50_ (mg/L)/MIC (mg/L).

Indirect hemolysis was performed following the above protocol, but using PBS supplemented with 2 mM CaCl_2_.

The hemagglutination assay was used to complement the previous study in order to determine the venom’s ability to induce erythrocyte aggregation. This assay was carried out according to the protocol described by Hae-Cho et al., [20], both in the presence and absence of CaCl_2_, since certain components of the venom, such as C-type lectins, may require Ca^2+^ to produce hemagglutination [17]. On the one hand, to perform the direct hemagglutination assay, the contents of each well of the hemolysis plate were resuspended with their corresponding pellet in the original Eppendorf tubes, and 200 µL/well was dispensed into a V-bottom plate (Thermo Fisher Scientific, Roskilde, Denmark). This was accompanied by a series of controls: a negative venom control, consisting of 180 µL of PBS and 20 µL of each venom concentration to be tested, and positive and negative hemagglutination controls, using Concanavalin A (final concentration of 1000 mg/L) and PBS, respectively. This plate was covered with parafilm and left to incubate at room temperature until the following day. Finally, hemagglutination was determined visually, with negative results observed as a compact pellet at the bottom of the well and positive results as a diffuse halo. On the other hand, indirect hemagglutination assays were performed in the same manner, using PBS supplemented with 2 mM CaCl_2_. These experiments were carried out in triplicate (*n* = 3).

### 4.8. Statistical Analysis

The results were expressed as mean ± standard deviation (SD) for biofilm formation inhibition, hemolysis, and hemagglutination data. Statistical analyses were performed using SPSS v20.0 (IBM Corp., Armonk, NY, USA). No formal statistical comparison was performed between the MIC, MBC, MBIC, and MBEC values of the three *S. aureus* strains (Table 1), given the limited number of clinical isolates tested (*n* = 2); these values are reported descriptively only. Differences in biofilm formation inhibition between venom-treated wells (1×, 2×, and 4× MIC) and untreated controls were evaluated for each of the three *S. aureus* strains using the independent experiment as the statistical unit. For each of the three independent experiments, the eight technical replicate wells were aggregated to a single background-subtracted median optical density value, yielding one paired control-treatment observation per experiment (*n* = 3 independent experiments per concentration and strain). Because control and treatment wells within a given concentration were processed in parallel on the same plate, paired Student’s *t*-tests were used to compare treatment vs. control values across the three independent experiments, with Benjamini–Hochberg correction applied across the nine pairwise comparisons performed. This approach was preferred over a mixed-effects model because reliable estimation of the random-effect variance requires substantially more than three independent experiments (clusters); with only three clusters available, a mixed model would not add information beyond this two-stage approach and could yield unstable estimates [43]. Differences in hemolysis and hemagglutination percentages between venom-treated concentrations and positive/negative controls (*n* = 3 per group) were evaluated using Student’s *t*-test, after confirming approximate normality via the Shapiro–Wilk test and homogeneity of variances. Statistical significance was established at *p* < 0.05.

## 5. Conclusions

This exploratory study provides preliminary evidence that captive-bred *B. rhinoceros* venom exerts potent bactericidal activity against planktonic MRSA isolates and inhibits early *S. aureus* biofilm formation, with a favorable hemocompatibility profile supporting its potential as a candidate source of topical antimicrobial compounds. Given these limitations, these findings should be regarded as hypothesis-generating rather than conclusive. As a specific next step, bioassay-guided fractionation of the venom by RP-HPLC is planned to isolate and test individual toxin fractions, with priority given to the PLA_2_ and LAAO fractions as the more mechanistically plausible contributors to antibacterial and anti-biofilm activity; the CTL fraction, despite its high relative abundance, will also be tested but remains a more speculative candidate given its typically non-enzymatic nature, alongside cytotoxicity screening in mammalian keratinocyte/fibroblast models to further assess translational potential.

## Figures and Tables

**Table 1:** Antibacterial and biofilm formation inhibitory properties of *Bitis rhinoceros*’ venom against the three strains of *S. aureus*.

Compound	Strain	MIC	MBC	MBIC	MBEC
*Bitis rhinoceros*’ venom	ATCC 29213	16	16	>1024	>1024
	MRSA 1	4	4	>1024	>1024
	MRSA 2	4	4	>1024	>1024

MIC: Minimum Inhibitory Concentration (mg/L); MBC: Minimum Bactericidal Concentration (mg/L); MBIC: Minimum Biofilm Inhibitory Concentration (mg/L); MBEC: Minimum Biofilm Eradication Concentration (mg/L). All assays were performed in quadruplicate (*n* = 4). Values reported as “>1024 mg/L” are censored values at the assay’s upper tested limit and do not represent precise measurements; the true MBIC/MBEC may lie above this threshold.

**Table 2:** Effect of the venom on the formation of *S. aureus* biofilms.

Compound	Strain	MIC	% Inhibition of Biofilm Formation at 1× MIC Mean ± SD (*n* = 3)	*p*-Value *	% Inhibition of Biofilm Formation at 2× MIC Mean ± SD (*n* = 3)	*p*-Value *	% Inhibition of Biofilm Formation at 4× MIC Mean ± SD (*n* = 3)	*p*-Value *
*Bitis rhinoceros*’ venom	ATCC 29213	16	99.43 ± 0.09	<0.001	99.73 ± 0.32	<0.001	99.68 ± 0.27	<0.001
	MRSA 1	4	98.86 ± 0.99	<0.001	98.65 ± 0.85	<0.001	98.96 ± 0.91	<0.001
	MRSA 2	4	97.85 ± 0.77	0.001	97.60 ± 0.88	0.001	97.08 ± 0.17	0.001

MIC: Minimum Inhibitory Concentration (mg/L); SD (standard deviation of the percentage of biofilm formation inhibition).

**Table 3:** Hematological properties of *Bitis rhinoceros* venom.

Compound	HC_50_	MIC ATCC 29213	MIC MRSA 1	MIC MRSA 2	SI ATCC 29213	SI MRSA 1	SI MRSA 2	*p*-Value
*Bitis rhinoceros*’ venom	>512	16	4	4	≥64	≥256	≥256	<0.001

HC_50_: 50% hemolytic concentration (mg/L); MIC: minimum inhibitory concentration (mg/L); SI: selectivity index (SI = HC_50_/MIC). The HC_50_ value is reported as “>512 mg/L”. For the purpose of calculating the SI, a concentration of 1024 mg/L was used when no hemolysis was observed at 512 mg/L, the highest concentration tested. Higher SI values indicate greater cellular selectivity.

## Data Availability

The mass spectrometry proteomics data have been deposited to the ProteomeXchange Consortium via the PRIDE partner repository under accession number PXD080981. All other data supporting the findings of this study are included within the article and its Supplementary Materials (Table S1).

## Supplementary Materials

The following supporting information can be downloaded at: https://www.mdpi.com/article/10.3390/antibiotics15100984/s1, Supplementary Table S1: MS/MS assignments of the toxins isolated from the venom of Bitis rhinoceros fractionated by RP-HPLC and SDS-PAGE.
